# Effect of leptin combined with CoCl_2_ on healing in Sprague Dawley Rat fracture model

**DOI:** 10.1038/srep30754

**Published:** 2016-07-29

**Authors:** Pengcheng Liu, Junfeng Liu, Kuo Xia, Liyang Chen, Xing Wu

**Affiliations:** 1 Department of Orthopedics, Shanghai Tenth People’s Hospital, School of Medicine, Tongji University, Shanghai 200072, P.R. China

## Abstract

To evaluate the effect of leptin combined with CoCl_2_ on rat femur fracture healing. 48 male Sprague Dawley rats were randomly divided into two main groups. Then standardized femur fractures were created to all rats. Control group rats were treated with 0.5 mL physiological saline, and experimental group rats were treated with 5 μg/Kg.d leptin and 15 mg/Kg.d CoCl_2_ along with 0.5 mL physiological saline for 42 days intraperitoneally. Each main group was divided into three subgroups for each evaluation at second, fourth and sixth weeks, each subgroup included eight rats. The radiological evaluation showed that the fracture healing progress of experimental group was superior to control group from second week. At fourth week, experimental group had better fracture healing progress than control group significantly. Results of biomechanics show the ultimate load (N) and deflection ultimate load (mm) of experimental group was significantly increased than that in control group from fourth week. The present result demonstrated that leptin combined with CoCl_2_ significantly increased the mRNA expression levels of HIF1A, Vegfa, Runx2, Bmp2, Bglap and Alpl. It suggested that leptin combined with CoCl_2_ have a positive effect on rat femur fracture healing by activating the HIF1A pathway.

Fractures are common in our daily life. In splintered fractures and diabetics populations, impaired fracture healing even bone defects frequently occur.

Leptin seems to play an important role in bone microstructural alterations and bone metabolism^1^,^2^. A recent meta-analysis suggested that leptin levels of human serum are positively associated with bone mineral content and bone mineral density^3^. Leptin has physiological functions such as maintaining bone mass to get better bone quality^4^. Recently, the important relationship between bone mass and leptin has gained popularity, but the role of leptin has not been fully elucidated^5^. Leptin plays an important role in the arrangement of the growth plate chondrocyte differentiation and the cartilage matrix maturation by secretion and side secretion mechanisms^6^. At the same time leptin effects differentiation and proliferation of osteoblasts and the coordination of bone development^7^. The effect of leptin on bone formation is not only as a systemic hormone, but also as the local factors in the formation of the vascular tissue of the cartilage^8^. Systemic application of leptin can directly affect the osteoblast and osteoclast, and reduce the brittleness of the bone^5^. Some studies report that the relationship between brain injury and fracture healing is linked to leptin^9^.

CoCl_2_ is a drug mimic for hypoxia, it can directly enhance HIF-1α stabilization by inhibiting prolyl hydroxylase enzymes^10^. Also, HIF-1α is well known involved in the impairment of fracture healing^11^. A recent animal study found that CoCl_2_ can induce bone and cartilage formation and increases vascularization, its function may through activating HIF-1α pathway^12^.

Many previous studies focus on the possible effects of leptin on angiogenesis, chondrocyte, and osteoblast differentiation. Recent studies also suggested that hypoxia is a powerful stimulus factor for fracture healing via the mediation of angiogenesis^12^. Our study aims at verifying whether leptin combined with CoCl_2_ play a significant positive role in accelerating healing progress of rat femur fracture.

## Materials and Methods

The research was carried out in the laboratory animal experimental center after the approval of the hospital ethics committee (Shanghai, China). The animal model is strictly in accordance with the guidelines of the Shanghai laboratory animal center and the policy of animal use in Shanghai Tenth People’s Hospital. This animal research protocol was approved by Animal Care and Welfare Committee of Tongji University. Forty-eight male Sprague Dawley rats provided by Shanghai SLAC Laboratory Animal Inc. with the standard of GB14924.2–GB14924.6, with a mean body weight of 371 g (range from 364 to 386 g) and a mean age of 9 weeks were equally and randomly divided into two main groups. Control group rats were treated with 0.5 mL physiological saline intraperitoneally every day as alone operative control group. Experiment group rats were treated with 5 μg/Kg.d leptin (Rat Recombinant Leptin, ProSpec, Rehovot, Israel) and 15 mg/Kg.d CoCl_2_ (Cobalt (II) chloride, hexahydrate, Sangon Biotech, Shanghai, China) within 0.5 mL physiological saline intraperitoneally every day. The day of surgery began treatment, and at the same time repeated daily for a total of 42 days. At the same time each main group rats were divided into three subgroups (n = 8 rats) for each evaluation at second, fourth and sixth weeks after the operation. We monitored the rats’ conditions every day and recorded.

### Femur fracture model

All rats were reared at the specific pathogen free laboratory animal experimental center, Shanghai Tenth People’s Hospital. After the adaptation period with free access to water and regular food of one week, all rats were fasted four hours before surgery. The operation was performed under anesthesia, and the method was intraperitoneal injection of 1% sodium pentobarbital (50 mg/kg body weight). The rats were placed supine and use 10% iodine solution to scrub the skin over the right femur preoperative and postoperative. All efforts were made to minimize their suffering.

A 1.0 mm stainless-steel rod was threaded directly into the medullary cavity of the rat right femur through the skin and patellar ligament over the rat knee and was finally advanced up to the distal end of the rat femur. The femur was exposed by the longitudinal median skin incision directly over the rat femur bone. Three holes were made at the right angles in the mid shaft of the rat femur by using a drill. Light manual bending gently broke the rat femur while a stainless-steel rod was held in right place in the medullary canal of femur. Then rat skin incision was closed with a 3–0 absorbable traction suture. All the operations were carried out by the same surgeon. After anesthesia, the rats were allowed to have full weight bearing and unrestricted movement. Preventative antibiotics were administered by intramuscular injection to all rats preoperatively and postoperatively. The rats were given free access to water and normal laboratory chow 6 hours after the operation.

Anesthetized rats were killed by cervical dislocation. Rat right leg was amputation from its hip joint. The rat femurs were stripped from their soft tissues and stored for radiological evaluation and biomechanical testing.

### Radiological evaluation

Radiological evaluation of Lane-Sandhu scoring system was used for bone formation in fracture healing progress^13^. The callus diameter of X-ray (mm) and volume of fracture callus (mm^3^) parameters were evaluated by two separate observers in a blinded manner according to the standard lateral radiographs which were taken after anesthesia. Serial micro-CT scans of these femur specimens were carried out after removing the intramedullary nail carefully under a micro-CT scanner (Bruker micro-CT 1076, Belgium) with protocols. For three dimensional (3-D) measurements, the volume of interest (VOI) was selected as 6 × 6 × 6 mm^3^ of fracture callus at the center of the fracture site. Then three dimensional (3-D) analysis was performed using the software CTAn (Bruker micro-CT, Belgium). The bone volume fraction (BV/TV) parameter was calculated, then the bone mineral density (BMD) was calibrated by using the attenuation coefficient s of two hydroxyapatite phantoms (supplied by Bruker micro-CT, Belgium) with defined BMD of 0.25 g/cm^3^ and 0.75 g/cm^3^.

### Histomorphometric evaluation

To evaluate new bone formation, double labeling on days 0 and 7 was used. On day 0, rats received subcutaneous injections of oxytetracycline hydrochloride (30 mg/kg) freshly dissolved in 0.2 ml of saline; and on day 7, rats received subcutaneous injections of calcein green (25 mg/kg) freshly dissolved in 0.2 ml of saline. Nine days after initiation of treatment, rats were terminated with an overdose of phenobarbital sodium. Tissue plugs corresponding to the micro-CT VOIs of femur plateaus were further processed for histological analysis. Serial sections were made into 5 mm thicknesses and microscopy of callus with oxytetracycline hydrochloride and calcein green bone double labeling fluorescence. An epifluorescence microscope (80i; Nikon, Brighton, Mich) (excitation wave lengths of 390 nm for oxytetracycline hydrochloride and 485 nm for calcein green) with a camera (Coolsnap K4; Photometrics, Tucson, Ariz) were used to capture fluorescent images of each histologic section. Labeling width was calculated as the average distance between the double-labels, and mineral apposition rate (MAR) was calculated by dividing the labeling width of the double labels by the interlabel time. Evaluations were conducted by an experienced expert blinded to the findings from X-ray and micro-CT. MAR was assessed by Image Pro-Plus 5.1 (Media Cybernetics, Silver Spring, MD, USA). Briefly, at least five sections from each sample were stained for analysis. For each section, five areas were measured.

### Biomechanical testing

All femora from rats were stored in separate sealed plastic bags at −20 °C after the radiological evaluation until one day before biomechanical testing through three-point bending by material testing system (858 Mini Bionix MTS Systems, Eden Prairie, Minnesota, USA). All femora were removed from the freezer and allowed to thaw overnight in the original sealed plastic bags before three-point bending testing. The intramedullary nail was removed before the three-point bending test carefully. After all pre-processing operations, the rat femora were tested with a 1-mm indenter, at a speed of 0.01 mm/s with a 15-mm span for femur. The test maintained until the breakage of femur occurred. Peak load and peak displacement were recorded by the data collector. Stiffness values were calculated by using those values. The ultimate load (N) and deflection ultimate load (mm) parameters were evaluated by an experienced expert blinded to the findings from radiological evaluation according to the curves of deflection position and fracture load force.

### Real-time PCR

Total RNA was extracted from fresh whole blood of each group using the TRIzol reagent (Invitrogen Life Technologies, Carlsbad, CA, USA). After RNA quantity and quality was determined then total RNA was reverse-transcribed. Real-time PCR was then performed to measure associated mRNA expression levels relative to GAPDH mRNA expression with the ABI PRISM^®^ 7500 Sequence Detection System (Applied Bio system, Foster City, CA, USA) using SYBR Green Master Mix (Toyobo, Co., Ltd.). Primers were synthesized by Sangon Biotech (Shanghai, China). The PCR thermal cycling conditions were according to the protocol. All experiments were performed in triplicate and were repeated three times. The Real-time PCR results were expressed relative to associated gene expression levels at the threshold cycle (Ct) and were related to the control.

### Statistical evaluation

All research results were expressed as means ± standard deviation (S.D.). The all available data were analyzed by SPSS statistical software version 21.0 as appropriate. Comparison between two-groups was analyzed by using Student t test if data were normally distributed. A Kruskal-Wallis test was carried out if data are not normally distributed followed by post-hoc comparison tests. Then a difference with *P* < 0.05 was usually considered to be significant statistically.

## Results

### Surgery results

Open transverse femur fractures were created in the experimental rat, and the fracture healing was typical secondary fracture healing. No complication occurred during anesthesia and intraperitoneal injection. Postoperative limping was only seen for first week, and then all rats could use their fixed extremities in normal fashion. No wound infection and no complication occurred. No rat died in the whole experiment. No statistically significant difference was observed in weight between the two groups. So the effect of weight on biomechanical results can be ignored.

### Radiological and histomorphometric results

**Figure 1:**
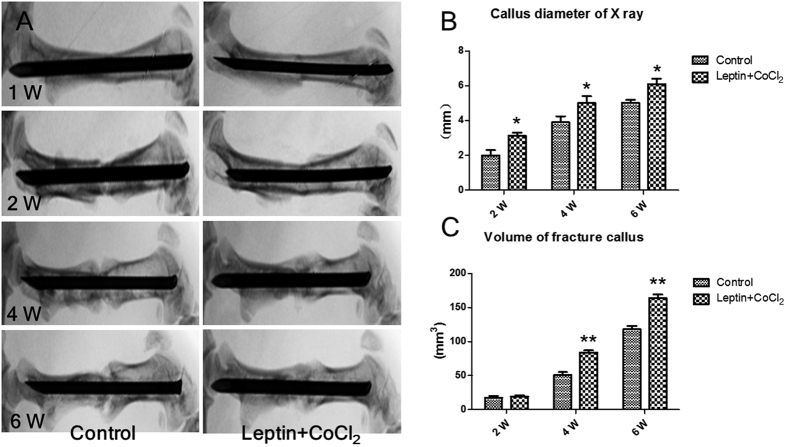
(**A**) Different degrees of union radiological images of two groups. (**B**) Callus diameter of X-ray (mm) of experimental group were larger than control group. (**C**) Volume of fracture callus (mm^3^) of experimental group were larger than control group. *P < 0.05; **P < 0.01 vs. Control group at indicated time.

**Figure 2:**
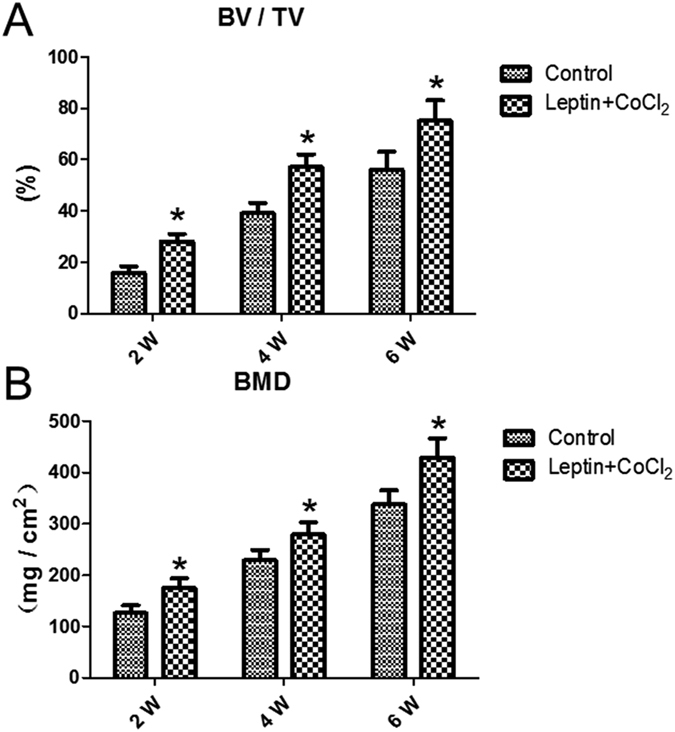
(**A**) Bone volume fraction (BV/TV) of experimental group were larger than that of control group from fourth week. (**B**) Bone mineral density (BMD) of the callus in the experimental group were significantly increased compared with control group from fourth week. *P < 0.05; **P < 0.01 vs. Control group at indicated time.

**Figure 3:**
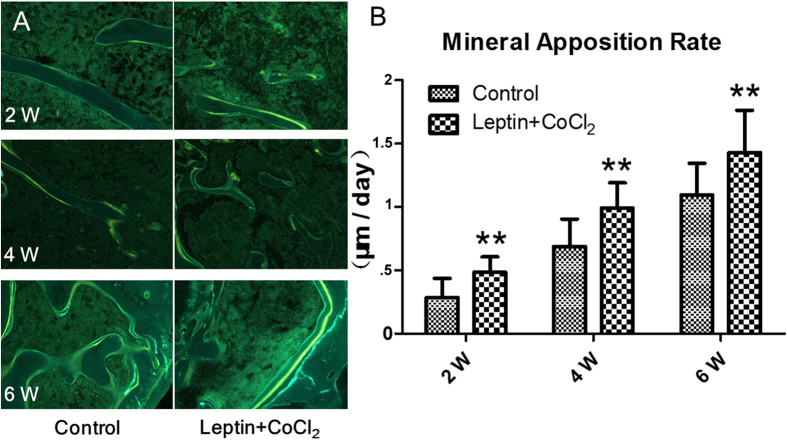
(**A**) Microscopy of callus with tetracycline bone double labeling fluorescence showed the growth distance of new bone between groups had significantly difference at sixth weeks. (**B**) Histomorphology showed the mineral apposition rate (MAR) between groups had significantly difference from fourth week. *P < 0.05; **P < 0.01 vs. Control group at indicated time.

Different degrees of fracture union progress in both group rats could be seen in Fig. 1. First week a full fracture line could be seen in both groups. There was no statistically significant difference observed between both groups at the first week. At second week the fracture lines in experiment group rats begin to disappear. At the second, fourth and sixth weeks, there was a statistically significant difference observed between both groups, fracture healing progress in experimental group rats were better than control group rats (*P* = 0.028, *P* = 0.019, *P* = 0.007, respectively) (Fig. 1A). The callus diameter of X-ray (mm) of experimental group were larger than control group and it had significantly different between two groups from second week (*P* < 0.05) (Fig. 2B); the volume of fracture callus (mm^3^) of experimental group were larger than control group and it had significantly different between two groups from fourth week (*P* < 0.01) (Fig. 1C). In both groups, there was no statistically significant difference observed between the second, fourth and sixth week in all subgroups respectively. Result of micro-CT scan showed that leptin combined with CoCl_2_ induced a larger and more maturated callus than control group, the cortical thick of callus is significantly exceeded that of control group, the healing processes were early than control group and the quality of callus were improved. The bone volume fraction (BV/TV) of experimental group rats were larger than those of control group rats from second week (*P* < 0.05) (Fig. 2A). Bone mineral density (BMD) of rat callus in the experimental group were significantly increased when compared with the control group from second week (*P* < 0.05) (Fig. 2B). Also, microscopy of callus with tetracycline bone double labeling fluorescence showed the growth distance of new bone between groups had significantly difference from second week (Fig. 3A), histomorphology showed the mineral apposition rate (MAR) between groups had significantly difference from second week (*P* < 0.01) (Fig. 3B).

### Biomechanical results

**Figure 4:**
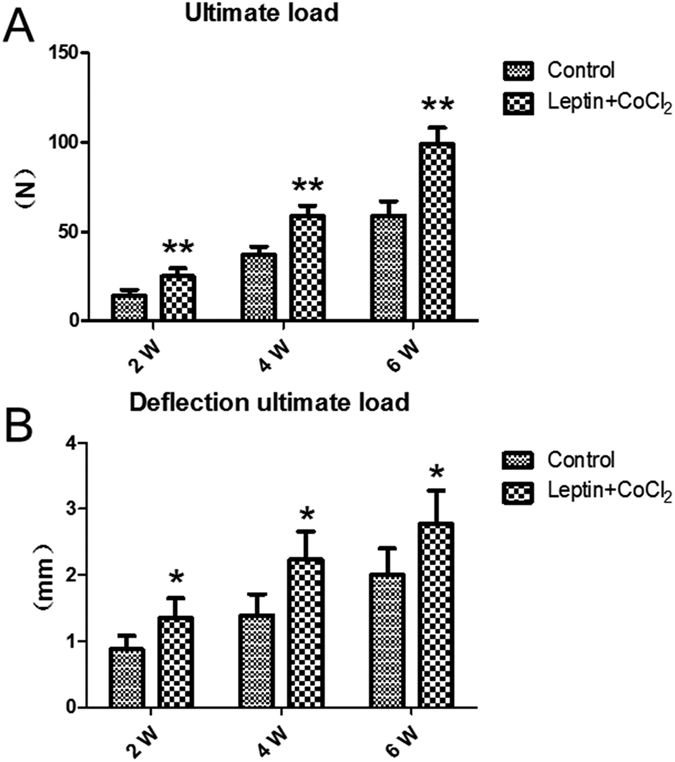
(**A**)Ultimate load (N) of experimental group was significantly increased than that of control group at fourth and sixth weeks. (**B**) Deflection ultimate load (mm) had significantly difference at fourth and sixth weeks. *P < 0.05; **P < 0.01 vs. Control group at indicated time.

Results of biomechanics show the ultimate load (N) of experimental group rats were significantly increased than those of control group rats from second week (*P* < 0.01) (Fig. 4A), the deflection ultimate load (mm) had significantly difference from second week too (*P* < 0.05) (Fig. 4B).

### Real-time PCR

**Figure 5:**
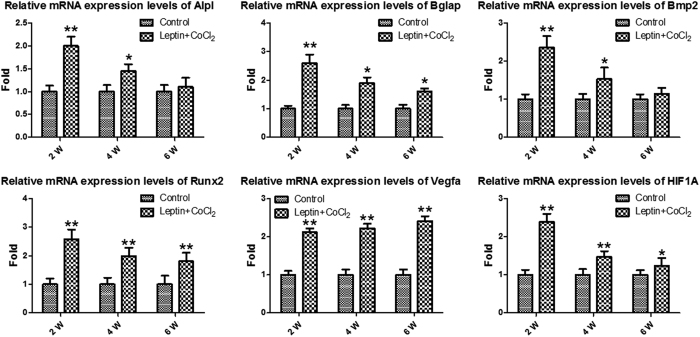
Relative mRNA expression levels of Alpl, Bglap, Bmp2, Runx2, Vegfa and HIF1A were determined by quantitative polymerase chain reaction. *P < 0.05; **P < 0.01 vs. Control group at indicated time. Alpl, Alkaline phosphatase, liver/bone/kidney; Bglap, Bone gamma-carboxyglutamate (gla) protein; Bmp2, Bone morphogenetic protein 2; Runx2, Runt-related transcription factor 2; Vegfa, Vascular endothelial growth factor A; HIF1A, hypoxia-inducible factor-1α.

As presented in Fig. 5, Rats with leptin and CoCl_2_ exhibited a significantly increased HIF1A expression at the measured time-points. To further evaluate whether HIF1A had a direct functional role in this process, the expression of downstream genes of HIF1A was investigated. The results also indicated that leptin combined with CoCl_2_ significantly increased vegfa, Runx2, Bmp2, Bglap and Alpl mRNA levels. These results suggested that the effect of leptin combined with CoCl_2_ on fracture healing partly involved the activation of the HIF1A signaling pathway.

## Discussion

With the development of the world’s population and technology, the frequency and severity of bone injury are increasing. It is a serious medicine problem for both doctors and patients. The research literature on fracture healing is also rapidly increasing^14^,^15^,^16^,^17^. A study from Beil FT *et.al.*^18^ indicated that the formation of bone has great significance in the various stages of fracture healing. A two fold speed increase in bone formation can significantly accelerate bone fracture healing process in the experimental mice. Therefore, increasing bone formation is an important way to improve fracture healing. Fracture healing is a process of wound healing, which is similar to bone growth and development, including the interaction between cells, growth factors and extracellular matrix. Inflammatory cells, vascular cells, bone progenitor cells and osteoclasts play an important role in the cellular level repair process. Similarly, proinflammatory cytokines, growth factors, proosteogenic factors and angiogenic factors also play a role in the molecular level of bone repair^19^,^20^.

The research studies about the effect of leptin and hypoxia on fracture healing is rapidly increasing. A recent study believed that leptin expression was in a unique stage of the process of fracture healing, the lack of leptin leads to delayed fracture healing and local application of leptin can accelerate fracture healing^21^. Some studies showed interesting results that leptin can accelerate fracture healing after traumatic brain injury^22^. Higher leptin levels are associated with increased bone formation in the fracture site^23^,^24^. A study from Kerimoglu G *et.al.*^25^ found that leptin has a dose-dependent positive effect on the healing of tibial fracture in rats. We observed the better fracture healing progress of the experiment group rats in this study.

Angiogenesis in the process of fracture healing provides nutrients, cells and biological media and waste disposal environment. Angiogenesis plays an important role in the process of osteogenesis^26^,^27^. These are the natural stages of fracture healing. MSCs can be recruited by osteocytes under hypoxia, and activation of HIF-1α increases angiogenesis. A study from Bouloumie *et al*.^28^ emphasized that leptin is a potent regulator of angiogenesis. At same time CoCl_2_ has an activation effect on migration of vascular endothelial cells and angiogenesis *in vivo*. In our study, leptin combined with CoCl_2_ could accelerate fracture healing significantly. Therefore, we believe that the effect of leptin combined with CoCl_2_ on angiogenesis may play an important role in the process of fracture healing.

Although the mechanism of leptin combined with CoCl_2_ promoting fracture healing is not clear, but we believe that the positive effects of leptin combined with CoCl_2_ in regulating angiogenesis in the cartilage into bone process, and promoting effects on osteogenic and chondrogenic differentiation and growth, and promoting effect on bone metabolism eventually led to this result.

In our study, we observed that leptin combined with CoCl_2_ have a positive effect on fracture healing, based on radiological evaluation results. The radiological outcome data from two groups was obtained without interruption, and showed the applicability of this method.

## Conclusion

In conclusion, intraperitoneal injection of leptin combined with CoCl_2_ has a positive effect on the healing of femoral fractures in rats. We conclude that leptin combined with CoCl_2_ can promote the formation of callus, and finally promote the fracture healing. The present study demonstrated that leptin combined with CoCl_2_ significantly increased the mRNA expression levels of HIF1A, Vegfa, Runx2, Bmp2, Bglap and Alpl, and the effect partly involved the activation of the HIF1A signaling pathway.

## Additional Information

**How to cite this article**: Liu, P. *et al*. Effect of leptin combined with CoCl_2_ on healing in Sprague Dawley Rat fracture model. *Sci. Rep.* **6**, 30754; doi: 10.1038/srep30754 (2016).
